# Mononuclear or Coordination Polymer Complexes? Both Are Possible for 3,6,9-Trioxaundecanedioic Acid

**DOI:** 10.3390/molecules28217410

**Published:** 2023-11-03

**Authors:** Giovanni Bella, Jan Holub, Giuseppe Bruno, Francesco Nicolò, Antonio Santoro

**Affiliations:** 1 Department of Chemical, Biological, Pharmaceutical and Environmental Sciences, University of Messina, Viale F. Stagno d’Alcontres 31, 98166 Messina, Italy; gbella@unime.it (G.B.); gbruno@unime.it (G.B.); fnicolo@unime.it (F.N.); 2 Department of Inorganic Chemistry, University of Chemistry and Technology, Prague, CZ-16628 Prague, Czech Republic; jan.holub@vscht.cz

**Keywords:** supramolecular chemistry, self-assembly, polymers, metal complexes

## Abstract

Investigating the driving forces leading to the formation of a specific supramolecular architecture among a wide spectrum of all the possibly obtainable structures is not an easy task. The contemporary literature provides several models for correctly predicting the thermodynamically accessible structures that can originate from a library of building blocks. Definitions are rigid by their very nature, so their application may sometimes require a shift in perspective. In the study presented herein, we describe the crystal structures of three metallo-supramolecular architectures assembled from deprotonated derivatives of 3,6,9-trioxaundecanedioic acid and Mn(II), Co(II) and Zn(II). In the Mn(II) case, the complexation resulted in a complex of a discrete/heptacoordinated nature, whereas the other two structures appeared as helical polymers. To explain such an anomaly, in this work, we describe how the interplay between the flexibility of the ligand spacer and the number of coordinating atoms involved determines the divergent or convergent organisation of the final coordination architecture.

## 1. Introduction

**Figure 1:**
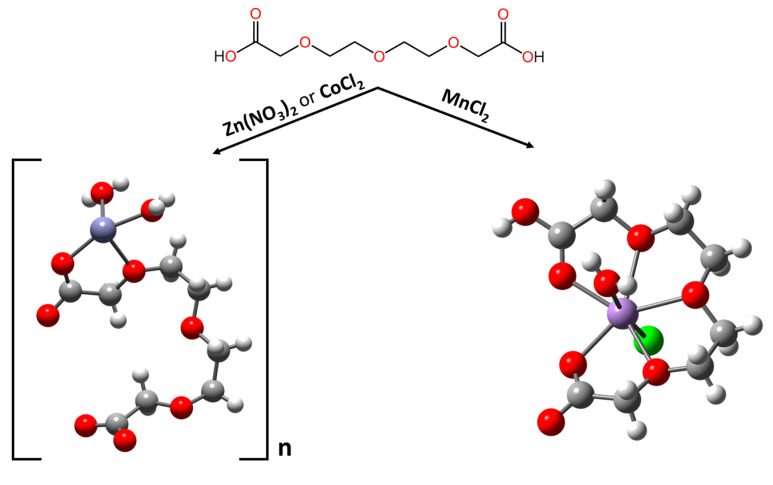
The structures of the three complexes discussed herein.

In the design of supramolecular systems, the nature of the interactions involved in the self-assembly process plays a prominent role in determining the structural and dynamic properties of the desired supramolecular assembly. Indeed, the spatial organisation and the reactive behaviour of the building blocks are strongly related to the nature, strength and directionality of the inter- and intramolecular interactions. To date, both organic and inorganic chemistries bring to the table a plethora of different possibilities for connecting the subunits of the whole system, ranging from hydrogen bonds [1,2], which are massively employed by nature in biological systems [3], and weak Van der Waals interactions [4] to coordination chemistry [5,6] and even some covalent, but still reversible, interactions [7,8,9,10]. Selecting the right interaction out of such a wide spectrum of possibilities to achieve the desired results can pose a difficult task. Despite this, in recent decades, coordination chemistry has demonstrated to be the most versatile choice to the self-assembly of topologically defined, 3D supramolecular structures [11,12,13,14,15,16]. The presence of different metal ions in these systems provides access to different coordination geometries, offering a rich range of patterns for connecting and spatially arranging organic ligands in pre-designed topological fashions [17,18]. When playing with binding strengths and preferential coordination geometries, the charges or ionic sizes of various metal ions allow one to control the spatial disposition of the components and define the dynamical features of the system through thermodynamic control. This provides access to complex assemblies that are practically inaccessible through other means and opens the gates to the design of (pre)programmed chemical systems [17]. The design and realisation of metallo-supramolecular architectures are being actively developed for a wide variety of purposes ranging from biomedical applications to nanotechnology and material science [19,20,21,22,23,24,25,26]. The programmed metallo-supramolecular self-organisation requires the encoding of suitable information (a programme) in the ligand strands. This is achieved via an appropriate choice of donor atoms, their spatial arrangement and connections, which all together define the molecular steric/spatial code of the self-organisation process [11,17,27]. The ligand’s code is then read/processed by the metal through its coordination predispositions, and the whole process is finally manifested in the form of a defined coordination architecture. This means that even for complex architectures, the assembly process is not accidental. It becomes possible to achieve different outputs (structures) by changing the reading algorithm (the set of metal ions) while maintaining the same information (the ligand). That being said, the role of the metal ions in such a system goes beyond the simple role of a template. In inorganic chemistry, although self-organisation includes a “templating” step, there is generally not one but several such steps that must act contemporaneously. A single template step requires only the information provided by the ligand’s binding unit and the metal’s geometric coordination preferences. However, in more complex self-organisation processes, additional information stored in the ligand’s backbone contributes to the spatial organisation of the binding sites of the ligands and the metal ions, thus providing an additional level of control over the shape of the final architecture [11]. The process described above is illustrated well via the formation of supramolecular grids [28,29,30,31,32], helicates [33,34,35,36,37] or knots [38,39,40,41,42]. In the first system, the rigidity of the ligand, in synergy with the position and spatial orientation of its binding sites, allows for the correct assembly of grid-shaped structures. The same binding sites with different spatial dispositions and a more flexible connection can lead to helicate structures. Herein, we present three different metallo-supramolecular architectures in a crystalline solid state. These three complexes comprise the same organic ligand, 3,6,9-trioxaundecanedioic acid, which coordinates three different metal ions, Co(II), Zn(II) and Mn(II) (Figure 1). The complexes obtained with CoCl_2_ and Zn(NO_3_)_2_ appear very similar in their crystalline states, showing a helical-shaped coordination polymer, while the complex containing MnCl_2_ shows a monometallic, nonpolymeric nature.

## 2. Structural Description

3,6,9-trioxaundecanedioic acid is a structurally flexible, polytopic ligand bearing seven possible coordination sites. The different functional groups that involve the oxygens determine the binding coordination preferences. This characteristic enhances the versatility of the ligand and creates the possibility to achieve a multitude of metal-based systems. In spite of the adaptability of our ligand, examples of it in stable, metal-based crystals are quite rare in the literature [43,44,45]. In such an unexplored landscape, we report three new crystal structures, analysed u single crystal X-ray diffraction, in which the combinations between the ligand and different metal centres give rise to different structural motifs.

Research on 3,6,9-trioxaundecanedioic acid complexes supports the capability of this polydentate ligand to act either in a bridging mode, interconnecting different metal centres, or in a chelating mode, folding around a single metal cation. The behaviour of this ligand can, to a certain extent, be influenced by the crystallization conditions, the metal atom and its respective coordination number. We obtained single crystals of three compounds suitable for X-ray analysis and investigated their structures to better understand the different dispositions of the ligand in different coordination setups/systems.

**Figure 2:**
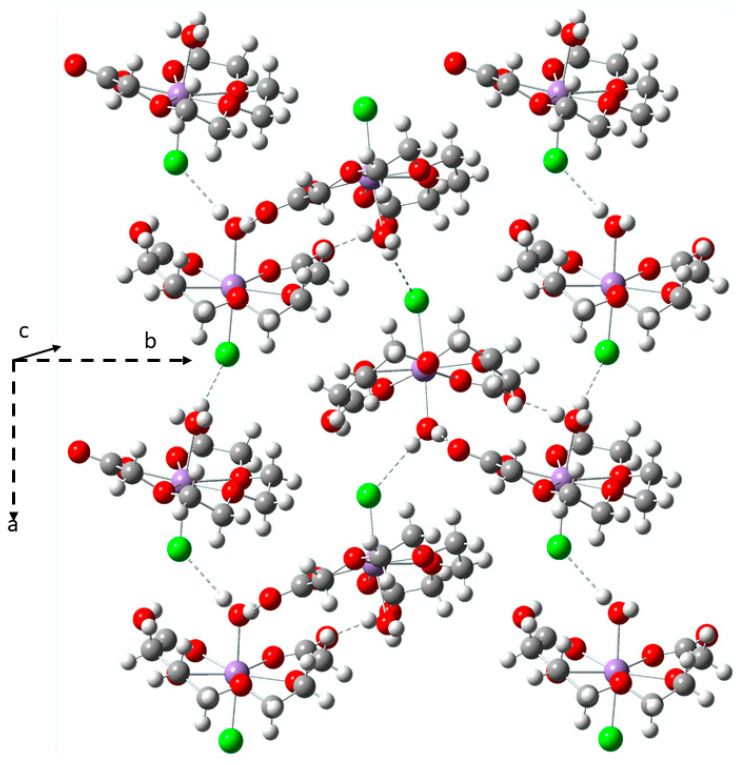
The crystal packing observed for the Mn(II) complex.

The mono-hydrogenated ligand derivative reacts with Mn(II) cations in a 1:1 ratio, and the corresponding crystal asymmetric unit comprises one discrete complex which is represented in Figure 2. In the crystal packing, the isolated molecules are interconnected by strong H bonds stemming from the two hydrogens of the coordinated water molecule that interact with a chlorine ion and a protonated carboxyl group. The Mn(II) ion shows a pentagonal, bipyramidal coordination with its axial positions occupied by chlorine and water, while the 3,6,9-trioxaundecanedioic acid with one protonated carboxylate lies on the equatorial plane. Its five oxygens (three oxygens from the ether functions, one -CO-O^-^ and one -COH=O) surround the centre of the Mn(II). This similar seven-coordination was already observed with more rigid and pre-organized ligands such as 15-crown-5 ether [46,47,48,49]. The distortion in the equatorial plane of our complex (evidenced by an O1-Mn-O5 angle of around 81.99°, shown in Table S2) when compared to the geometry shown in the 15-crown-5 ether complexes, in which the angle involving two adjacent coordination atoms on the equatorial plane and the central metal ion ranges from around 71 to 73° [46], can be attributed to the looser, more open conformation of 3,6,9-trioxaundecanedioic acid.

**Figure 3:**
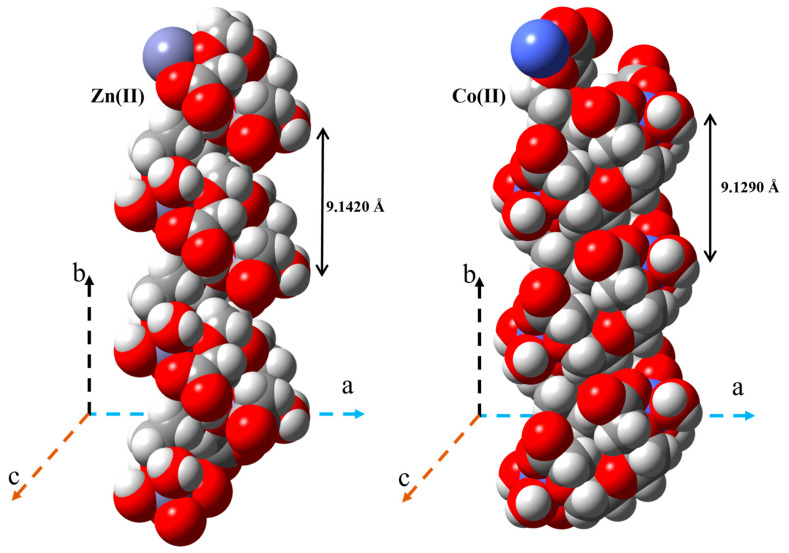
Helical structures of the Co(II) and Zn(II) complexes obtained via SC-XRD; the reported distances describe the respective helix pitches (expressed in Angstrom).

The ligand reacts with Zn(II) and Co(II) ions in a 1:1 ratio and produces solid-state polymeric crystals which are quite isomorphous (Figure 3), with the corresponding atoms’ coordinates related by an approximate “x, 1 − y, z” transformation. Their asymmetric unit contains one octahedral metal atom linked by two fully deprotonated 3,6,9-trioxaundecanedioic acid ligands to the adjacent metal in “−1/2 − x, −1/2 + y, 3/2 − z” and “−1/2 − x, 1/2 + y, 3/2 − z” equivalent positions, generating a polymeric helix around the crystallographic 21-screw axis. This column is chiral, but the crystallographic centres of symmetry create a racemic mixture of the opposite linear screws. The centrosymmetric crystal packing is formed by these helicoidal cylinders parallel to the b-axis and interconnected by a strong H-bond network. This network involves the hydrogens of both of the two coordinated aqua ligands as well as the H_2_O molecules interspersed in the crystal lattice. The metal cation shows a distorted octahedral geometry mainly due to the small chelating bite, shortening the corresponding coordination angles O2-M-O3 (76.83 and 76.65° for Zn(II) and Co(II), respectively) and O5-M-O7 (75.42 and 76.15° for Zn(II) and Co(II)) of the two ligand bridges, as evidenced by the data reported in Table S1.

## 3. Discussion

**Scheme 1:**
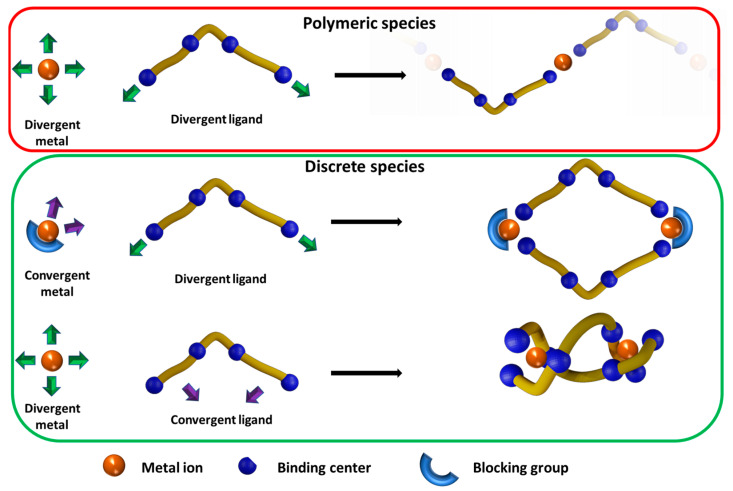
The possible design of discrete and polymeric metallo-supramolecular architectures.

To shed light on the reasons that lead to the differences in shape and crystal packing between the Co(II), Zn(II) and Mn(II) complexes, it is worth pointing out the concepts of host–guest chemistry and divergent/convergent binding sites. Paraphrasing Donald J. Cram, in host–guest chemistry, the host component consists of an organic species in which the binding site converges in the complex. At the same time, the guest consists of a species in which the binding sites diverge in the complex [50]. Within the context of metallo-supramolecular aggregates, Makoto Fujita pointed out that the nature of the output, be it polymeric or discrete, can be successfully predicted by relying on the nature of the building blocks utilised [26,51,52]. Specifically, Fujita’s approach is based on the concept of the divergent/convergent binding sites of both the ligand and the metal ion. As illustrated in Scheme 1, a metallo-supramolecular polymer is obtained only if both the metal ion and the ligand involved possess divergent binding sites (described in the red box in Scheme 1). In the other situations (green box) in which at least one of the ligand and the metal ion or both of them possess convergent binding sites, the self-assembly process results in a discrete supramolecular complex. In this regard, one could call polymeric self-assembly a recessive characteristic.

**Figure 4:**
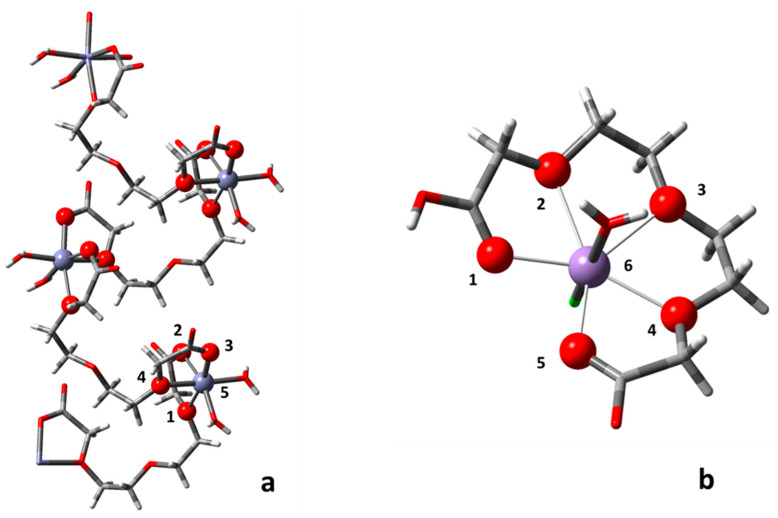
Coordination geometries of (**a**) Co(II) and Zn(II) and (**b**) Mn(II) complexes.

A discrete structure may be composed of very few components, considering a convergent host (ligand) that completely envelops a divergent guest. Conversely, it may also comprise a higher number of components if the host can’t be enveloped and several centres are bridged by the ligand. In the majority of cases, the guest metal ion presents divergent binding sites. The exceptions to this are metal ions in which two or more of the adjacent binding sites are irreversibly coordinated [52,53,54] (the blocking group in Scheme 1). All our complexes fall under the category of divergently binding metal cations; therefore, considering that the complexes were obtained under the same experimental conditions, the different natures of the observed complexes are attributable only to the different metal cation used (the reading algorithm of the self-assembly process). The convergent/divergent nature of a binding site does not depend only on the “denticity” of the site itself but also strongly on the spatial predispositions of the ligand (e.g., its rigidity) and metal (e.g., its coordination preference). There are many examples in the literature of polytopic ligands with bidentate or tridentate divergent binding sites that can form metallo-supramolecular polymers with a suitable set of metal ions [55,56]. To the best of our knowledge, the case presented here is a rare example [44] in which a ligand can form polymeric or discrete structures in the presence of different but still divergent metal ions. As shown in Figure 4a, the Zn(II) metal ion in the helical complex is coordinated by 3,6,9-trioxaundecanedioic acid through the carboxylic oxygens (oxygens 2 and 3) and the adjacent etheric ones (oxygens 1 and 4), thus providing two bidentate binding sites. The result is a ligand flexible enough to spatially arrange the coordination subunits in a divergent way, allowing for a polymeric structure. The same considerations valid for the Zn(II) helical complex are also applicable to the Co(II) polymeric structure.

As far as the Mn(II) complex is concerned (showed in Figure 4b), the coordination geometry imposed by the Mn(II) metal ion, which is approximately 10% bigger in size than Zn(II), allows the trioxaundecanedioic acid to envelop a single metal ion by using a total of five oxygens, three coming from the etheric chain (oxygens 2, 3 and 4 in Figure 4b) and two from two different carboxylic groups (oxygens 1 and 5). Heptacoordination in metal complexes involving the first row of the transition metal series is relatively rare, but the heptacoordinated Mn(II)-based structures are more abundant than the other metal ions in the same series [57,58]. What sets our complex apart is the fact that unlike the pre-organized ligands reported thus far [46,47,48,49], our ligand possesses complete conformational freedom. The “pentadentate” ligand could be hypothetically and conveniently considered a combination of a tridentate binding site (i.e., where oxygens 1, 2, and 3 act as coordination atoms) and a bidentate binding site (where oxygens 4 and 5 operate for coordination). Following this logic, the spacer that connects these two subunits consists of only two sp3 carbon atoms. Differing from the longer spacer backbone seen in the helical complexes, the length of the spacer in the latter case is simply not long and flexible enough to confer divergent binding sites to the trioxaundecanedioic acid. Such a condition then leads to the discrete complex being the most favoured structure and the only observed product of the self-assembly process.

## 4. Materials and Methods

The chemicals and solvents used were purchased from commercial suppliers (Sigma Aldrich, Merk Life Science S.r.l., Milan, Italy) and were used without further purification.

The Zn(II) complex was achieved through the reaction of one equivalent of Zn(NO_3_)_2_·6H_2_O (around 4.5 × 10^−5^ mol; 13.4 mg) with the same equivalent of 3,6,9-trioxaundecanedioic acid (4.5 × 10^−5^ mol; 10 mg) in few ml of water. The mixture was stirred for 1 h and then evaporated under reduced pressure. As a result, colourless crystals were obtained. A similar procedure was used to obtain the Co(II) and Mn(II) complexes, using one equivalent of CoCl_2_ (pink solid crystals) or MnCl_2_, respectively.

Suitable crystals were selected, and their diffraction data were collected at room temperature using a single crystal Bruker APEX-II CCD diffractometer and the APEX2 package [59,60], which was used for data reduction and structure solutions (SHELXT [61]), while refinement was carried out using the wlsqr technique (based on F2) of SHELXL [62] in OLEX2 [63], which also prepared the publication material.

The non-hydrogen atoms were refined anisotropically, while the alkyl H atoms were introduced in calculated positions and their bond geometry and isotropic displacement parameters were constrained to ride on their parent atoms. The hydrogens of the coordinated water molecules were located on the final ∆F map and refined isotropically. In both the Zn(II) and Co(II) compounds, the crystallization water showed an obvious rotational disorder, and its hydrogens were omitted due to the lack of any reasonable interpretation of their significant electron density residuals surrounding the O atom.

Select information about the crystal parameters and structural determination is provided in Table 1. Copies of the data can be obtained free of charge via an application to CCDC, 12 Union Road, Cambridge, CB2 1EZ, UK (fax: (internat.) 1 44-1223/336-033; e-mail: deposit@ccdc.cam.ac.uk), by using the deposition numbers 2268292/2268293/2268294 for the Zn/Co/Mn compounds, respectively.

All the other crystallographic data of the three compounds are reported in the Supplementary Materials.

## 5. Conclusions

Three new metallo-supramolecular complexes were obtained and characterised in a solid state with the aid of X-ray diffraction spectrometry. An in-depth description of the structural parameters of these complexes was presented. The described species differ due to the presence of different metal ions (Co(II), Zn(II) and Mn(II)), though they share the same organic ligand, 3,6,9-trioxaundecanedioic acid. Nevertheless, the crystal structures analysed showed pronounced topological differences, giving rise to two distinct architectures, i.e., two polymeric species (the Zn(II) and Co(II) complexes) and a discrete system (Mn(II) complex). To explain the unusual divergence in the nature of the supramolecular architectures, several considerations regarding the underlying steric reasons driving the evolution of the investigated systems into discrete or polymeric species were proposed. Our reasoning is based on the fact that in all three complexes, the organic ligand shows a divergent or convergent binding nature in response to the changes in its flexibility caused by the different numbers of binding sites involved in the coordination of the metal ions, which is an effect of the different algorithms (metal ions) used to read and process the information encoded in the ligand strand.

## Figures and Tables

**Table 1:** Crystal and refinement data for the three 3,6,9-trioxaundecanedioic acid complexes.

	Zn(II) Complex	Co(II) Complex	Mn(II) Complex
CCDC number	2268292	2268293	2268294
Empirical formula	C_8_H_18_O_10_Zn	C_8_H_18_O_10_Co	C_8_H_15_ClO_8_Mn
Formula weight	339.59	333.15	329.59
Temperature [K]	296 (2)	296 (2)	296 (2)
Crystal system	monoclinic	monoclinic	orthorhombic
Space group (number)	$P2_{1}/n$ (14)	$P2_{1}/n$ (14)	$Pna2_{1}$ (33)
a [Å]	10.1034 (2)	10.0374 (5)	13.772 (5)
b [Å]	9.1415 (2)	9.1290 (5)	11.737 (4)
c [Å]	14.8364 (3)	14.9490 (8)	7.707 (2)
α [°]	90	90	90
β [°]	96.6700 (10)	97.200 (3)	90
γ [°]	90	90	90
Volume [Å^3^]	1361.02 (5)	1359.0 (1)	1245.8 (7)
Z	4	4	4
ρcalc [gcm^−3^]	1.657	1.628	1.757
μ [mm^−1^]	1.848	1.306	1.303
F(000)	704	692	676
Crystal size [mm^3^]	0.38 × 0.33 × 0.23	0.40 × 0.31 × 0.27	0.34 × 0.29 × 0.20
Crystal colour	colorless	pink	colorless
Crystal shape	irregular	irregular	prismatic
Radiation	MoKα (λ = 0.71073 Å)	MoKα (λ = 0.71073 Å)	MoKα (λ = 0.71069 Å)
2θ range [°]	5.17 to 62.33 (0.69 Å)	5.24 to 60.29 (0.71 Å)	6.32 to 56.16 (0.75 Å)
Index ranges	−14 ≤ h ≤ 14 −13 ≤ k ≤ 13 −21 ≤ l ≤ 21	−14 ≤ h ≤ 14 −12 ≤ k ≤ 12 −21 ≤ l ≤ 21	−18 ≤ h ≤ 18 −15 ≤ k ≤ 15 −10 ≤ l ≤ 10
Reflections collected	60962	56629	67968
Independent reflections	4389 R_int_ = 0.0230 R_sigma_ = 0.0099	3995 R_int_ = 0.0319 R_sigma_ = 0.0147	3023 R_int_ = 0.0452 R_sigma_ = 0.0164
Completeness	99.8%	99.8%	99.8%
Data/restraints/parameters	4389/0/185	3995/0/188	3023/1/175
Goodness-of-fit on F^2^	1.065	1.079	1.044
Final R indexes [I ≥ 2σ(I)]	R_1_ = 0.0241 wR_2_ = 0.0694	R_1_ = 0.0267 wR_2_ = 0.0723	R_1_ = 0.0193 wR_2_ = 0.0416
Final R indexes [all data]	R_1_ = 0.0290 wR_2_ = 0.0730	R_1_ = 0.0352 wR_2_ = 0.0768	R_1_ = 0.0239 wR_2_ = 0.0429
Largest peak/hole [eÅ^−3^]	0.79/−0.28	0.70/−0.25	0.20/−0.15
Flack X parameter	---	---	0.020 (6)

## Data Availability

Crystals details are provided in the supporting information file.

## Supplementary Materials

The supporting information can be downloaded at: https://www.mdpi.com/article/10.3390/molecules28217410/s1, Figure S1. Ortep view of one asymmetric unit of the two isomorphous Zn(II) and Co(II) polymeric crystals. Displacement ellipsoids are drawn at 50% probability level while hydrogen size is arbitrary. Figure S2. View of the Mn(II) complex with the protonated ligand. Displacement ellipsoids are drawn at 50% probability level while hydrogen size is arbitrary. Figure S3. Zn and Co crystal packing showing the helix column parallel to b-axis and interconnected by the hydrogen bond involving the crystallization waters. Atoms are drawn at 50% probability level while hydrogen size is arbitrary. Table S1. Selected distances [Å] and angles [°] for Zn and Co complexes (1st and 2nd values, respectively). Table S2. Selected lengths [Å] and angles [°] for Mn TODD complex.
