# Supplementary material for: Mononuclear or Coordination Polymer Complexes? Both Are Possible for 3,6,9-Trioxaundecanedioic Acid
Source: Molecules. 2023 Nov 3;28(21):7410. doi: 10.3390/molecules28217410 (PMC10650274; doi:10.3390/molecules28217410)
Supplement: Supplementary file 1 [file molecules-28-07410-s001.zip › molecules-2588221-supplementary.pdf]

Monunuclear or coordination polymer complexes? Both are possible for 3,6,9-trioxaundecanedioic acid.

Giovanni Bella,[1] Jan Holub,[2] Giuseppe Bruno,[1] Francesco Nicolò[1] and Antonio Santoro*[1]

[1] Department of Chemical, Biological, Pharmaceutical and Environmental Sciences, University of Messina, viale F. Stagno d'Alcontres 31, 98166 Messina, Italy.
[2] Department of Inorganic Chemistry, University of Chemistry and Technology, Prague, CZ-16628 Prague, Czech Republic.
* Correspondence: antonio.santoro@unime.it

…………………………………………………………………………………………………….

…………………………………………………………………………………………………….

…………………………………………………………………………………………………….

…………………………………………………………………………………………………….

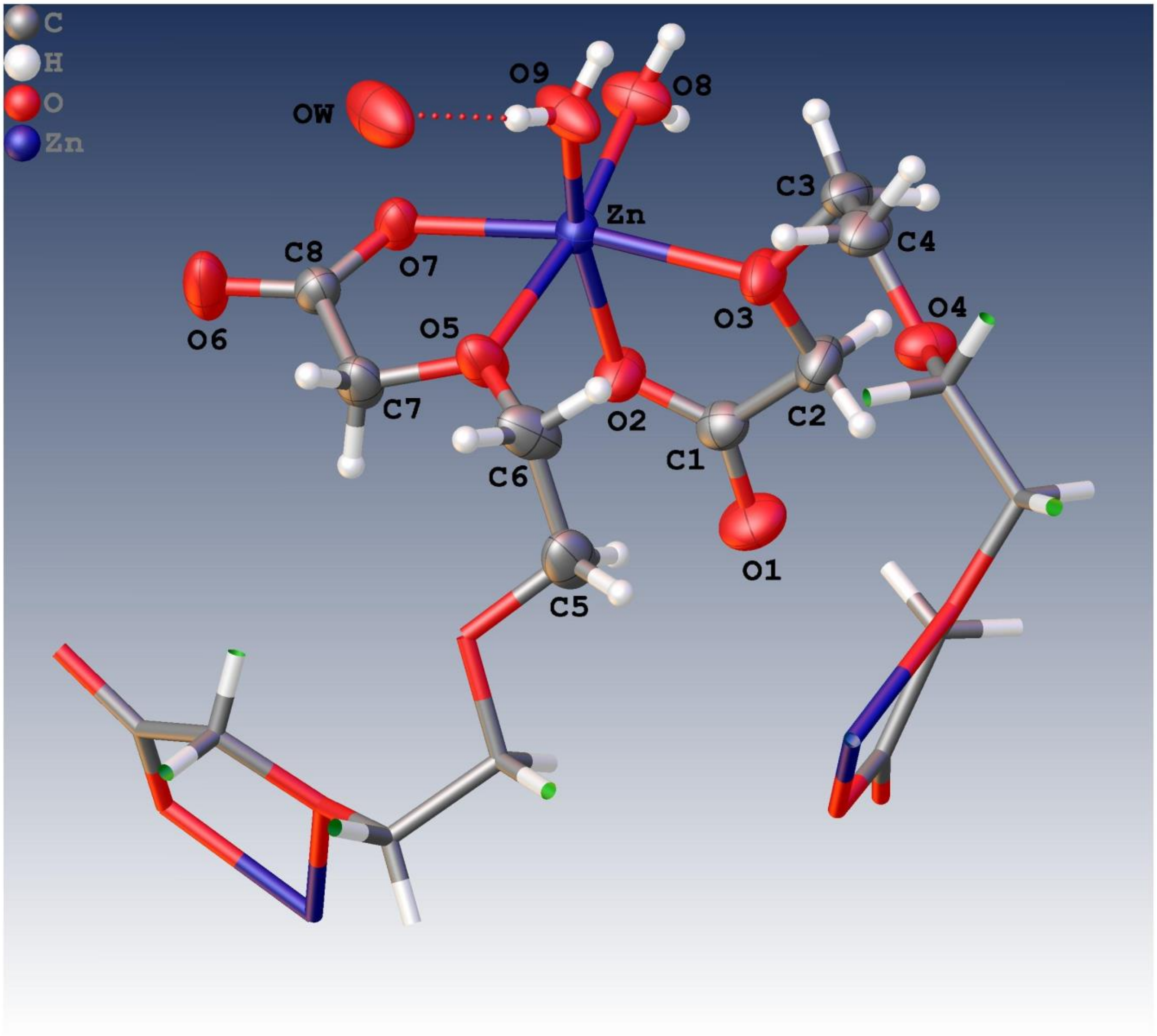

Figure S1. Ortep view of one asymmetric unit of the two isomorphous Zn(II) and Co(II) polymeric crystals. Displacement ellipsoids are drawn at 50% probability level while hydrogen size is arbitrary.

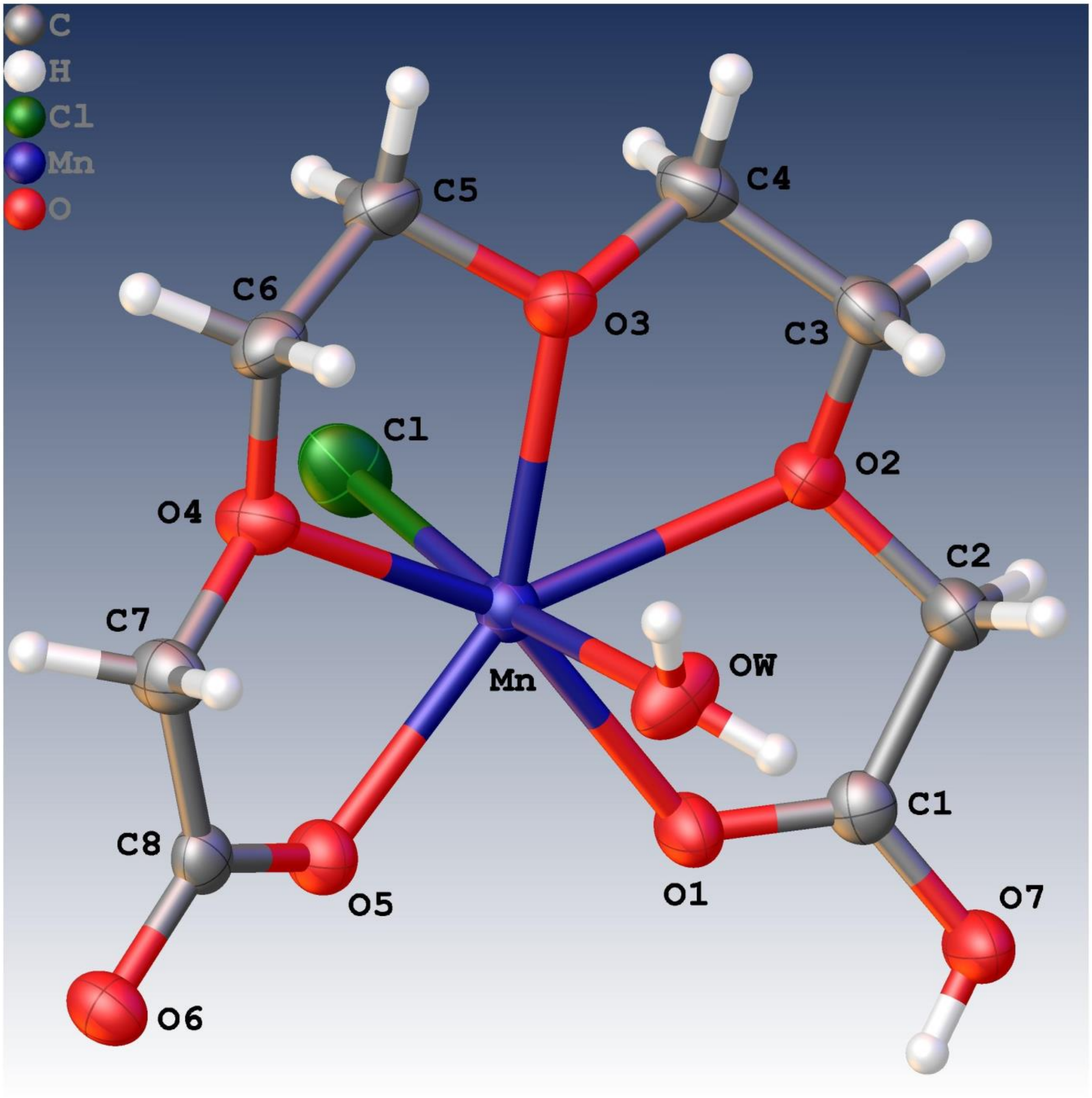

Figure S2. View of the Mn(II) complex with the protonated ligand. Displacement ellipsoids are drawn at 50% probability level while hydrogen size is arbitrary.

Table S1. Selected distances [Å] and angles [°] for Zn and Co complexes
(1st and 2nd values, respectively).

M–O2	2.016(1)	2.022(1)	M–O3	2.179(1)	2.168(1)
M–O5	2.225(1)	2.170(1)	M–O7	2.041(1)	2.047(1)
M–O8	2.037(1)	2.066(1)	M–O9	2.004(1)	2.004(1)
O2–M–O3	76.83(4)	76.65(4)	O2–M–O5	88.50(5)	87.94(5)
O2–M–O7	95.16(4)	95.36(5)	O2–M–O8	96.27(5)	96.09(5)
O3–M–O5	96.29(4)	99.58(5)	O7–M–O3	168.77(4)	171.19(4)
O7–M–O5	75.42(4)	76.15(4)	O8–M–O3	92.51(5)	89.26(5)
O8–M–O5	170.76(5)	170.94(5)	O8–M–O7	96.22(5)	95.35(5)
O9–M–O2	162.82(6)	163.47(6)	O9–M–O3	88.32(5)	89.62(5)
O9–M–O5	84.55(5)	85.28(5)	O9–M–O7	98.25(5)	97.62(6)
O9–M–O8	92.92(5)	92.86(6)			

D–H···A [Å]	d(D–H) [Å]		d(H···A) [Å]		d(D···A) [Å]		<(DHA) [°]	
O8–H8A···O1$^{\#1}$	0.79(2)	0.81(3)	1.96(2)	1.95(3)	2.732(2)	2.738(2)	164(2)	164(3)
O8–H8B···O6$^{\#2}$	0.83(2)	0.84(3)	1.89(2)	1.89(3)	2.712(2)	2.726(2)	175(2)	179(2)
O9–H9A···O7$^{\#2}$	0.74(2)	0.76(3)	1.98(3)	1.97(3)	2.721(2)	2.724(2)	175(3)	173(2)
O9–H9B···OW	0.71(2)	0.72(2)	1.92(2)	1.92(2)	2.631(2)	2.638(2)	174(3)	176(2)

Symmetry transformations used to generate equivalent atoms:
#1: -X, -Y, 2-Z; #2: 0.5-X, 0.5+Y, 1.5-Z;

Table S2. Selected lengths [Å] and angles [°] for Mn TODD complex**.**

Mn–Cl	2.450(1)	Mn–OW	2.219(2)	Mn–O1	2.217(2)
Mn–O2	2.404(1)	Mn–O3	2.339(2)	Mn–O4	2.286(2)
Mn–O5	2.177(1)				

O1–Mn–Cl	99.65(5)	O2–Mn–Cl	90.08(4)
O3–Mn–Cl	92.70(4)	O4–Mn–Cl	91.62(4)
O5–Mn–Cl	99.39(5)	O1–Mn–OW	83.20(7)
O2–Mn–OW	84.35(6)	O3–Mn–OW	80.43(6)
O4–Mn–OW	89.03(6)	O5–Mn–OW	88.01(6)
Cl–Mn–OW	172.34(5)	O1–Mn–O2	68.33(6)
O2–Mn–O3	69.41(6)	O3–Mn–O4	68.67(5)
O4–Mn–O5	70.49(6)	O5–Mn–O1	81.99(6)

C2–C1	1.497(3)	O1–C1	1.216(2)	O7–C1	1.296(3)
C7–C8	1.507(3)	O5–C8	1.239(2)	O6–C8	1.272(2)

O1–C1–C2	121.8(2)	O7–C1–C2	114.6(2)	O1–C1–O7	123.6(2)
O5–C8–C7	119.4(2)	O6–C8–C7	117.4(2)	O5–C8–O6	123.2(2)

O2–C2–C1–O1	−4.1(3)	O3–C4–C3–O2	60.8(2)
O3–C5–C6–O4	−54.4(2)	O5–C8–C7–O4	11.8(3)

D–H···A [Å]	d(D–H) [Å]	d(H···A) [Å]	d(D···A) [Å]	<(DHA) [°]
OW–HWB···O6[#1]	0.85(4)	1.89(5)	2.737(3)	170(3)
O7–H7···O6[#2]	0.82(4)	1.69(4)	2.512(2)	179(4)

Symmetry transformations used to generate equivalent atoms:
#1: 1-X, 2-Y, 0.5+Z; …….#2: 1-X, 2-Y, -0.5+Z

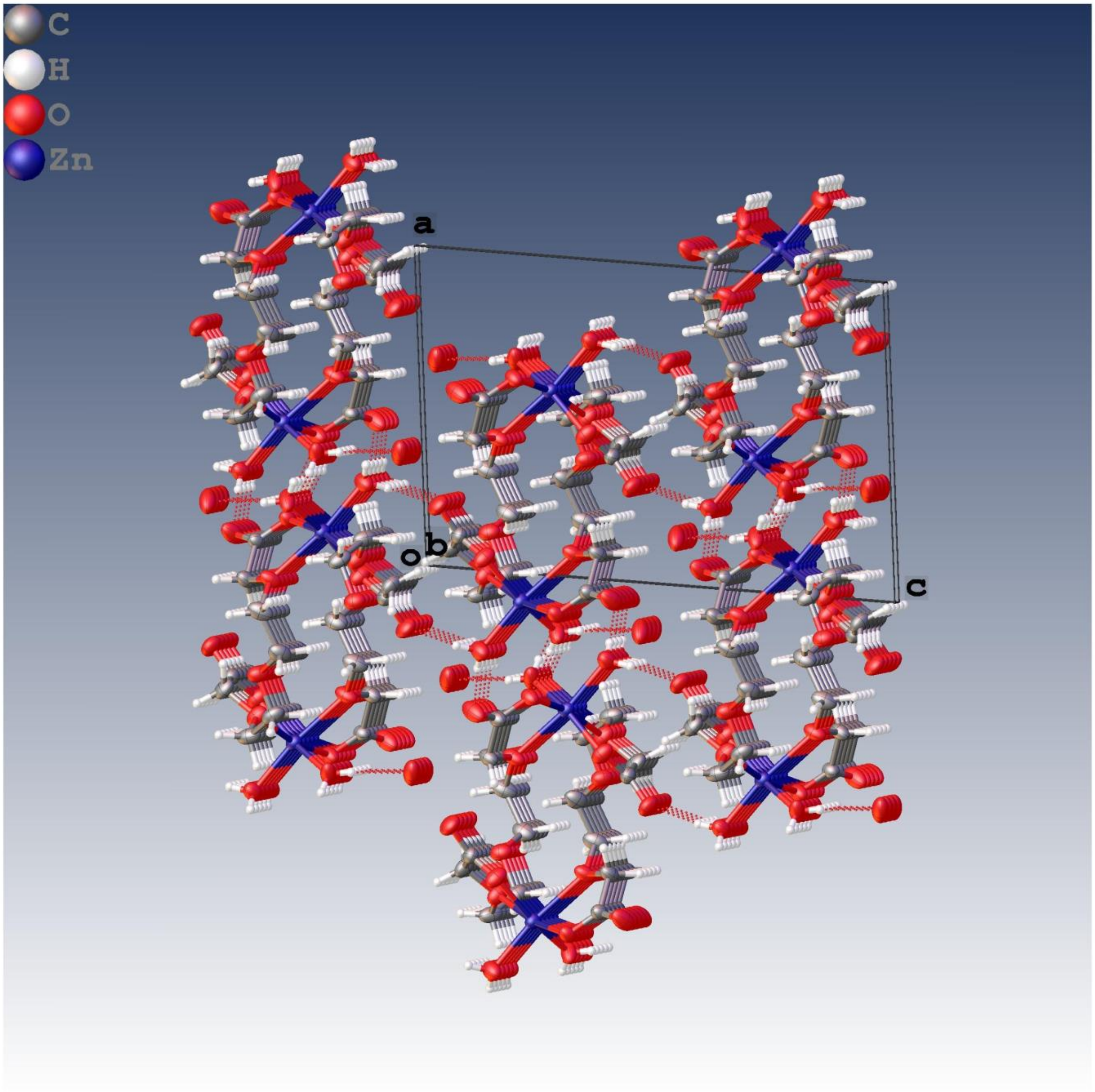

Figure S3. Zn and Co crystal packing showing the helix column parallel to b-axis and interconnected by the hydrogen bond involving the crystallization waters. Atoms are drawn at 50% probability level while hydrogen size is arbitrary.
